# Low cognitive functioning and depressive symptoms in patients with rheumatoid arthritis and systemic sclerosis: a clinical study

**DOI:** 10.1186/s12888-023-04995-3

**Published:** 2023-07-18

**Authors:** Panagiotis Alexopoulos, Maria Skondra, Marina Charalampopoulou, Eliza Eleni-Zacharoula Georgiou, Antonios Alexandros Demertzis, Suzana Ιoanna Aligianni, Philippos Gourzis, Antonios Politis, Polychronis Εconomou, Dimitrios Daoussis

**Affiliations:** 1 Mental Health Services, Department of Medicine, School of Health Sciences, Patras University Hospital, University of Patras, Rion 26504 Patras, Greece; 2 Global Brain Health Institute, School of Medicine, Trinity College Dublin, University of Dublin, Dublin, Republic of Ireland; 3 Department of Psychiatry and Psychotherapy, Klinikum rechts der Isar, Faculty of Medicine, Technical University of Munich, Munich, Germany; 4 First Department of Psychiatry, Eginition Hospital, School of Medicine, National and Kapodistrian University of Athens, Athens, Greece; 5 Department of Psychiatry, Division of Geriatric Psychiatry and Neuropsychiatry, Johns Hopkins Medical School, Baltimore, USA; 6 Department of Civil Engineering (Statistics), School of Engineering, University of Patras, Patras, Greece; 7 Department of Rheumatology, University of Patras Medical School, Patras, Greece

**Keywords:** Short-term memory, Concentration/attention, Verbal fluency, Inductive reasoning

## Abstract

**Background:**

Recently, cognitive deficits occurring in rheumatic diseases have attracted scientific attention. Cognitive symptoms in patients with Rheumatoid Arthritis (RA) and Systemic Sclerosis (SSc) have not been thoroughly studied. This study aimed to assess cognitive function and its relationship with depressive symptoms in RA and SSc and compare it to mild neurocognitive disorder due to Alzheimer’s disease (MiND) and to individuals without cognitive impairment.

**Methods:**

Cognitive function and depressive symptoms were tapped with the Cognitive Telephone Screening Instrument plus (COGTEL+), the Serial Seven Test (SST), the Mini-Mental State Examination (MMSE) and the Geriatric Depression scale-15 (GDS), respectively. Statistical analyses included between groups-, correlation- and regression analyses. Demographic characteristics were considered in the regression models.

**Results:**

The study included 30 individuals with RA, 24 with SSc, 26 adults without cognitive impairment and 33 individuals with MiND. Lower performance in verbal short-term memory, concentration/attention, verbal fluency and MMSE in patients with RA compared to individuals without cognitive impairment was detected. Of note, performance on verbal fluency, concentration/attention, inductive reasoning and MMSE was lower in RA compared to MiND. Individuals with SSc performed worse in verbal fluency and in MMSE in comparison to adults without cognitive deficits. Verbal fluency deficits in SSc exceeded that in MiND. Performance on MMSE, COGTEL+, prospective memory, working memory, verbal fluency and concentration/attention was related to GDS scores, which did not vary across the groups.

**Conclusions:**

Patients with RA and SSc encountered cognitive dysfunction, which partially pertains to depressive symptoms. Of note, the severity of cognitive dysfunction in many cases exceeded that of MiND.

## Background

Rheumatoid arthritis (RA) and Systemic sclerosis (SSc) are systemic autoimmune rheumatic diseases. The former approximately affects 1% of the global population, while the latter with a global prevalence of approximately 17.6 per 100.000 is less common [1–3]. Regarding their phenotypes, RA usually presents as symmetric polyarthritis of the hands and feet. Even though the main system affected is the musculoskeletal, RA is a systemic disease and may pertain to extra-articular manifestations specifically in patients with autoantibodies (rheumatoid factor or antibodies against citroulinated peptides) [1]. SSc is a systemic rheumatic disease which is associated with progressive thickening of skin, starting from the fingers in the form of sclerodactyly, and with fibrosis of internal organs (e.g. interstitial lung disease) [2, 3].

Cognitive deficits and depressive symptoms have been reported in individuals suffering from RA or SSc. Cognitive dysfunction in RA has a prevalence ranging from 38 to 71% and is mainly reflected in difficulties with divided/sustained attention, learning, memory, inhibition, mental flexibility, executive functions and visuo-spatial processing [4–6]. Furthermore, cognitive impairment is observed in 8.47–65% of patients with SSc [7–9] and is characterized by decreased performance in visual-spatial- and problem-solving abilities, as well as by poor attention and memory [10, 11]. Depression is substantially more common in RA than in the general population and its prevalence ranges from 14 to 48% [12, 13]. Prevalence of depression among patients with SSc ranges between 16.2% and 68.4% [14–17]. The vulnerability of patients with RA and SSc to developing depression may be related to pain, physical disability, diminished quality of life, poor social support, emotion-focused coping, helplessness, fear of progression as well as to potential side effects from disease- modifying antirheumatic drugs and glucocorticoids [18–20].

Although RA and SSc have been related to cognitive impairment, potential differences in their cognitive phenotypes as well as the associations of depressive symptoms with cognitive function have not been thoroughly investigated yet. The aims of the present study were (i) to study cognitive function and its relationship with depressive symptoms in patients with RA or SSc, (ii) to compare it to patients with mild neurocognitive disorder due to Alzheimer’s disease (MiND), an oligosymptomatic stage of Alzheimer’s disease [21] negatively affecting performance in complex activities of daily living and quality of life [22], and to individuals without cognitive impairment.

## Materials and methods

### Participants

Patients suffering from SSc and RA, who attended follow-up appointments at the outpatient clinic of the Department of Rheumatology of the Patras University Hospital between January and September 2019, and patients with MiND and individuals without cognitive impairment who were assessed at the psychogeriatric outpatient clinic of the Department of Psychiatry of the above hospital between January and July 2021 (convenience sample) were asked to participate in the study. Inclusion criteria for the entire sample were (i) diagnosis of RA, SSc, MiND or absence of both neurocognitive- and rheumatic disorders and (ii) treatment/assessment at outpatient units of Patras University Hospital. Exclusion criteria were (i) diagnosis of major neurocognitive disorder (ii) diagnosis of MiND caused by a disease other than Alzheimer’s disease (e.g. frontotemporal lobar degeneration, Parkinson’s disease), (iii) diagnosis of a rheumatic disease other than RA or SSc, (iv) coexistence of RA or SSc with MiND, (v) mental or neurological disorder or unstable medical condition potentially affecting cognitive function (e.g. major depression, schizophrenia, multiple sclerosis, seizure disorder, head injury, uncontrolled hypothyroidism), (vi) hearing or visual difficulties, being potential sources of bias in diagnostic accuracy, (vii) insufficient knowledge of the Greek language (viii) unwillingness to participate in the study. RA was diagnosed according to the updated classification criteria published in 2010 by the American College of Rheumatology (ACR) and European League Against Rheumatism (EULAR) [23]. SSc diagnosis was based on the 2013 ACR/EULAR classification criteria [24], while the diagnosis of MiND relied on the DSM-5 diagnostic criteria [25] and on the guidelines of the National Institute on Aging- Alzheimer Association [26]. In individuals without cognitive impairment, neither cognitive deficits nor functional impairment were detected. The study was conducted in accordance with the latest revision of the Declaration of Helsinki and was approved by the Bioethics and Research Ethics Committee of the University of Patras (Approval number: 45,156/2017). Written informed consent was obtained from all participants.

### RA and SSc related characteristics and treatment

The description of clinical phenotypes and treatment of patients with RA and SSc was based on several parameters. Disease activity was assessed in patients with RA with the Disease Activity Score 28 (DAS28) [1]. Anti-citrullinated protein/peptide antibody, rheumatoid factor and radiographic erosions were also recorded [1]. The former two are markers of seropositivity, while the latter illustrates erosive bone damage. In patients with SSc, physical function was measured with the Disability Index and the Scleroderma-Specific Health Assessment Questionnaire (SHAQ) [2, 3]. The SHAQ combines the disability and pain scales of the HAQ with five scleroderma-specific visual analogues scales (VASs) for digital ulcers, Raynaud’s phenomenon, gastrointestinal symptoms, lung symptoms, and overall disease severity, with each VAS score scaled from 0 to 3. In addition, in patients with SSc we also recorded the following markers: (i) autoantibodies such as anti-centromere or anti-topoisomerase I, (ii) the modified Rodnan skin score, mirroring skin thickness, (iii) pulmonary function tests including forced vital capacity, and the diffusing capacity of the lungs for carbon monoxide, both widely used to monitor SSc-related interstitial lung disease (ILD), (iv) system involvement such as the presence of ILD, SSc related pulmonary arterial hypertension, gastrointestinal manifestations, digital ulcers and/or SSc related muscle disease [2, 3]. Finally, treatment with steroids, classic disease-modifying anti-rheumatic drugs, such as methotrexate and leflunomide, hydroxychloroquine, other immunosuppressants such as azathioprine and mycophenolate mofetil, targeted biologic therapies and vasoreactive therapies with bosentan and sildenafil were recorded in detail [1–3].

#### Assessment of cognitive function and depressive symptoms

Cognitive function was assessed with the Cognitive Telephone Screening Instrument plus (COGTEL+), the Serial Seven Test (SST) and the Mini Mental State Examination (MMSE), while depressive symptoms were tapped with the Geriatric Depression scale-15 (GDS) [27–30]. COGTEL + is a brief test battery. It assesses prospective memory, i.e. the memory for intentions (0 or 1 point) [31], verbal short- and long-term memory (0–8 points each), working memory (0–12 points), which refers to mechanisms and processes that hold the mental representations currently most needed for an ongoing cognitive task available for processing [32], verbal fluency (0 to unlimited; as many words as the participant can name within 1 min), inductive reasoning (0–8 points), which is defined as ‘reasoning’ from particular cases to general principles [33], and temporal- and spatial orientation (0–6 points). The scores of the seven subtests are combined in the form of a weighted total score (7.2×prospective memory + 1.0×verbal short-term memory + 0.9×verbal long-term memory + 0.8×working memory + 0.2×verbal fluency + 1.7×inductive reasoning score + orientation) [30]. COGTEL + can be administered both in face-to-face sessions and over the telephone and the administration modality does not significantly affect participant performance [30]. The SST was employed to measure auditory attention/concentration, mental tracking and computation [29]. MMSE is a widely used albeit hardly sensitive brief tool in detecting mild cognitive deficits [28]. Furthermore, GDS is a brief instrument for screening, evaluating and diagnosing depressive symptoms and its items require a yes/no response [27]. Of note, GDS-15 does not include items related to the somatic symptoms of depression, which could be present in individuals with rheumatic diseases even in the absence of depression and subsequently embody a source of bias [34].

### Statistical analyses

Data normal distribution was tested with the Shapiro-Wilk W test. Demographic, clinical and cognitive performance differences were studied with one way analysis of variance (ANOVA) Kruskal–Wallis, Wilcoxon rank-sum (Mann-Whitney) test or chi-square test as appropriate. Post hoc comparisons were performed using Bonferroni post-hoc for the ANOVA, Dunn’s post-hoc test for the Kruskal–Wallis case and adjusted residuals and Bonferroni correction for the chi-square test. Relationships between depressive symptoms and cognitive function on the one side and DAS28- or SHAQ- score and treatment on the other were investigated with the Spearman rank-order correlation coefficient. Stepwise linear, logistic and ordered logistic regression models were employed for studying the relationship between both cognitive function and depressive symptoms, which were included in the models as dependent variables, diagnostic status, demographic (age, sex, education)- and clinical data, which were the independent variables.

## Results

The study included 30 consecutive individuals with RA, 24 with SSc, 26 adults without cognitive impairment and 33 individuals with MiND. The demographic and clinical characteristics of the four groups are shown in Table 1. The groups differed with regard to sex distribution, age and education (Table 1). Of note, no differences were detected in demographic characteristics between patients with SSc and RA. According to Spearman rank-order correlation coefficient, DAS28 score pertained in patients with RA to working memory (-0.408, P = 0.031), long-term memory (-0.429, P = 0.023), COGTEL + scores (-0.463, P = 0.016) and MMSE (-0.383, P = 0.045) indicating that high disease activity is inversely associated with cognitive function. Furthermore, DAS28 was positively associated with GDS scores (0.422, P = 0.025) pointing to a link between disease activity and depression. Treatment with steroids was inversely related to long-term memory (-0.415, P = 0.022) as well as to COGTEL + total score (-0.377, P = 0.04). In SSc, disease severity as assessed with SHAQ was inversely related to short-term memory (-0.566, P = 0.004) and verbal fluency performance (-0.502, P = 0.02). Treatment with vasoreactive agents (bosentan and/or sildenafil) was found to be inversely associated with prospective memory (-0.580, P = 0.003), long-term memory (-0.597, P = 0.002), working memory (-0.435, P = 0.034) and COGTEL + total scores (-0.525, P = 0.008).

**Table 1:** Demographic and clinical data of study groups

	Individuals without cognitive impairment (Group 1, G1)	Mild neurocognitive disorder due to Alzheimer’s disease (Group 2, G2)	Rheumatoid Arthritis (Group 3, G3)		Systemic Sclerosis (Group 4, G4)		Pairwise comparisons						
							G1 vs. G2	G1 vs. G3	G1 vs. G4	G2 vs. G3	G2 vs. G4	G3 vs. G4	
N	26	33	30		24								
Age, years*	62.81 (9.69) [43–79]	73.09 (7.18) [58–86]	63.23 (11.01) [29–82]		60.54 (10.14) [36–77]		< 0.001^‡‡^	1.000^‡‡^	1.000^‡‡^	< 0.001^‡‡^	< 0.001^‡‡^	0.904^‡‡^	
Education, years*	13.42 (2.35) [9–16]	9.79 (4.37) [4–18]	9.13 (4.21) [3–18]		10.04 (4.31) [6–18]		< 0.003^‡‡^	0.001^‡‡^	0.017^‡‡^	1.000^‡‡^	1.000^‡‡^	1.000^‡‡^	
Sex (female, N, %)	17 (65.38)	15 (45.45)	23 (76.67)		19 (79.17)		0.518^†^	0.189^†^	0.558^†^	0.009^†^	0.014^†^	1.00^†^	
***Demographic and clinical data***													
Disease duration, years*	N/A	N/A	13.85 (9.10) [2–28]		11.38 (7.86) [1–28]							0.349^‡^	
	N/A	N/A	DAS 28*	3.09 (1.34) [1.11-6.00]	SHAQ*	1.5 (0.66) [0–2]							
			RF, (N)	15	Diffuse Systemic sclerosis (N)	12							
			ACPA (N)	10	Limited Systemic sclerosis (N)	12							
			Erosions (N)	11	ATA (N)	12							
					ACA (N)	6							
					mRSS*	6.35 (4.54) [2–18]							
					FVC*	88.13 (17.14) [62–119]							
					DLCO*	59.76 (17.89) [21–97]							
					ILD (N)	18							
					GI (N)	17							
					DU (N)	13							
					MUSC (N)	3							
					PAH (N)	1							
***Antirheumatic treatment***													
Steroids (N)			17		3								
HCQ (N)			6		4								
AZA (N)			0		1								
MMF (N)			0		8								
DMARD (MTX or LEF) (N)			21		0								
Biologictherapies (N)			16		8								
Vasoreactive therapy (BOS and/orPDE5i) (N)			0		10								

*mean (standard deviation)[range];

DAS28: Disease Activity Score 28; RF: rheumatoid factor; ACPA: Anti-citrullinated peptide antibody; SHAQ: Scleroderma Health Assessment Questionnaire; ATAAnti-topoisomerase I antibodies; ACA :anti-centromere antibodies mRSS: modified Rodnan skin score; FVC: Forced vital capacity; DLCO: Diffusing capacity of the lungs for carbon monoxide; ILD: Interstitial lung disease; GI: Gastrointestinal manifestations; DU: Digital ulcers; MUSC: SSc related muscle disease; PAH: Pulmonary arterial hypertension; HCQ: Hydroxychloroquine, AZA: Azathioprine, MMF: Mycophenolate mofetil, DMARDs: Disease-modifying anti-rheumatic drugs; BOSbosentan PDE5i: phosphodiesterase 5 inhibitors

^‡‡^Dunn’s post-hoc test p-value after Kruskal–Wallis test (Bonferroni adjustment), ^‡^Wilcoxol rank-sum (Mann-Whitney) test; ^†^Pearson Chi square post hoc using adjusted residuals and Bonferroni correction

**Fig. 1:**
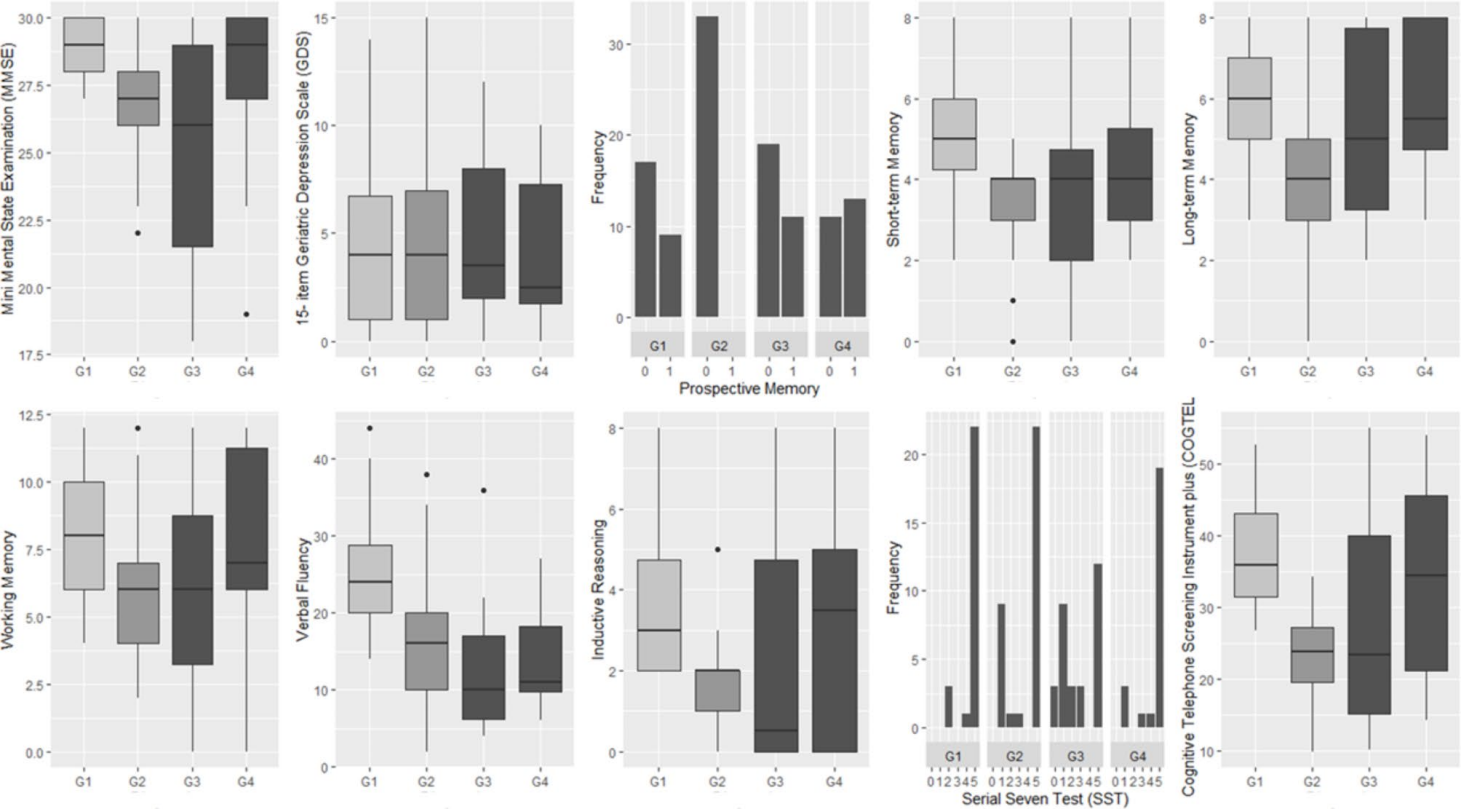
Performance on cognitive instruments and the 15-item Geriatric depression scale of individuals without cognitive impairment (G1), participants with Mild Neurocognitive Disorder (G2), and individuals with either Rheumatoid Arthritis (G3) or Systemic Sclerosis (G4)

Cognitive function was related to the diagnoses of RA and SSc as well as to depressive symptoms. Group comparisons unveiled significant differences in several cognitive domains between patients with RA and individuals without cognitive deficits, while only performance on verbal fluency tasks significantly differed between patients with SSc and adults without cognitive impairment (Table 2; Fig. 1). The final selected stepwise regression models with different cognitive domains as dependent variable and age, sex, education, diagnostic status and GDS scores as independent variables unveiled that compared to individuals without cognitive deficits, patients with RA exhibited lower performance in verbal short-term memory, verbal fluency, concentration/attention and in MMSE, while the difference in both inductive reasoning and COGTEL + scores tended to attain statistical significance (Table 3). The performance of patients with SSc was lower in verbal fluency and MMSE in comparison to adults without cognitive deficits. Verbal fluency performance was lower in RA than in SSc and in both groups lower than in patients with MiND (Tables 2 and 3). Individuals with MiND performed worse on short- and long- term memory, verbal fluency, as well as on MMSE and COGTEL + compared to cognitively healthy individuals (Table 3). Of note, the magnitude of the impact of RA on MMSE, verbal fluency, inductive reasoning and attention/concentration was higher than that of MiND (Table 3). In addition, the impact of SSc on verbal fluency exceeded that of MiND (Table 3). Depressive symptoms, which did not vary across the groups (Table 2), were inversely related to prospective memory, working memory, verbal fluency, concentration/attention, COGTEL + total score and MMSE, while the association between GDS score and performance on inductive reasoning task tended to attain statistical significance (Table 3), reflecting the interrelations between depressive symptoms and cognitive deficits.

**Table 2:** Cognitive performance and depressive symptoms in study groups

	Individuals without cognitive impairment (Group 1, G1)	Mild neurocognitive disorder due to Alzheimer’s disease (Group 2, G2)	Rheumatoid Arthritis (Group 3, G3)	Systemic Sclerosis (Group 4, G4)	Pairwise comparisons					
					G1 vs. G2	G1 vs. G3	G1 vs. G4	G2 vs. G3	G2 vs. G4	G3 vs. G4
MMSE*	29.00 (1.06) [27–30]	26.70 (2.05) [22–30]	25.40 (3.57) [18–30]	27.75 (2.72) [19–30]	< 0.001^‡‡^	< 0.001^‡‡^	0.264^‡‡^	1.000^‡‡^	0.064^‡‡^	0.0176^‡‡^
GDS*	4.69 (4.57) [0–14]	5.42 (4.71) [0–15]	4.77 (3.76) [0–12]	4.13 (3.46) [0–10]	1.0000^‡‡^	1.000^‡‡^	1.000^‡‡^	1.000^‡‡^	1.000^‡^	1.000^‡‡^
Prospective Memory*	0.35 (0.49) [0–1]	0 (0) [0–0]	0.37 (0.49) [0–1]	0.54 (0.51) [0–1]	0.012^‡‡^	1.000^‡‡^	0.391^‡‡^	0.004^‡‡^	< 0.001^‡‡^	0.485^‡‡^
Short-term Memory*	5.19 (1.36) [2–8]	3.30 (1.19) [0–5]	3.53 (2.16) [0–8]	4.46 (1.72) [2–8]	< 0.001^‡^	0.002^‡^	0.705^‡^	1.000^‡^	< 0.060^‡^	0.254^‡^
Long-term Memory*	5.96 (1.37) [3–8]	3.73 (1.68) [0–8]	5.23 (2.16) [2–8]	6.04 (1.81) [3–8]	< 0.001^‡^	0.786^‡^	1.000^‡^	0.007^‡^	< 0.001^‡^	0.608^‡^
Working Memory*	7.88 (2.60) [4–12]	5.61 (2.33) [2–12]	6.33 (3.34) [0–12]	7.79 (3.45) [0–12]	0.023^‡^	0.307^‡^	1.000^‡^	1.000^‡^	0.039^‡^	0.434^‡^
Verbal Fluency*	25.62 (7.63) [14–44]	15.33 (7.79) [2–38]	12.40 (7.07) [4–36]	13.79 (5.91) [6–27]	< 0.001^‡‡^	< 0.001^‡‡^	< 0.001^‡‡^	0.2819^‡^	1.000^‡‡^	0.997^‡‡^
Inductive Reasoning*	3.65 (1.92) [2–8]	1.79 (1.36) [0–5]	2.33 (3.04) [0–8]	3.21 (2.87) [0–8]	0.004^‡‡^	0.007^‡‡^	0.670^‡‡^	1.000^‡‡^	0.202^‡‡^	0.247^‡‡^
COGTEL+*	37.19 (7.39) [26.7–52.6]	23.27 (5.56) [9.90–34.30]	28.33 (14.57) [10.2–55.0]	34.20 (13.28) [14.3–54.0]	< 0.001^‡‡^	0.003^‡‡^	0.538^‡‡^	0.448^‡‡^	0.004^‡‡^	0.196^‡‡^
SST*	4.62 (0.98) [2–5]	3.76 (1.82) [1–5]	2.80 (1.97) [0–5]	4.38 (1.38) [1–5]	0.238^‡‡^	< 0.001^‡‡^	1.000^‡‡^	0.079^‡‡^	0.739^‡‡^	0.004^‡‡^

*mean (standard deviation)[range];

MMSE: Mini-Mental State Examination; GDS: Geriatric Depression scale; SST: Serial Seven Test; COGTEL+: Cognitive Telephone Screening Instrument plus;

^‡‡^Dunn’s post-hoc test p-value after Kruskal–Wallis test (Bonferroni adjustment), ^‡^ Bonferroni post-hoc test after ANOVA

**Table 3:** Demographic (age, sex, education) and clinical characteristics affecting cognitive function and depressive symptoms according to final selected regression models

Independent Variables	MMSE^‡^	GDS^‡^	Prospective Memory ^‡‡^	Short-term Memory^‡^	Long-term Memory^‡^	Working Memory^‡^	Verbal Fluency^‡^	Inductive Reasoning^‡^	SST^‡^	COGTEL+^‡‡‡^
MiND	-1.219**	0.809*		-1.300***	-1.216***		-1.581****			-4.901**
RA	-2.392****			-1.283***			-3.263****	-0.776*	-1.437***	-3.799*
SSc	-0.838****						-2.960****			
Sex		0.799**	1.604**	0.633*				-0.721*	-0.834	
Age, years	-0.062***	-0.030	-0.135****		-0.076****	-0.077****	-0.058***	-0.091****		-0.434****
Education, years	0.091*	-0.065	0.167**	0.199***	0.153****	0.168****	0.121***	0.249****	0.234****	1.040****
GDS	-0.094**		-0.186**			-0.129***	-0.119***	-0.081*	-0.136**	-0.558***

MiND: Minor Neurocognitive Disorder due to Alzheimer’s Disease; RA: Rheumatoid Arthritis, SSc: systemic Sclerosis; GDS: Geriatric Depression Scale; MMSE: Mini Mental State Examination, SST: Serial Seven Test; COGTEL+: Cognitive Telephone Screening Instrument plus

Stepwise regression models (alpha to enter 0.15, alpha to remove 0.16).

Empty cells point to variables not included in the final selected regression models.

‡ Ordered logistic regression

‡‡ Logistic regression

‡‡‡ Multiple linear regression

∗significant at 0.1 level, ∗∗significant at 0.05 level, ∗∗∗significant at 0.01 level, ∗∗∗∗significant at 0.001 level

## Discussion

The present study sheds light on cognitive performance and its relationship with depressive symptoms in individuals suffering from RA or SSc compared to individuals without cognitive deficits and people with MiND. The novelty of the study comprises (i) the evaluation of cognitive domains with a COGTEL + capturing interindividual differences in cognition across the full range of adult cognitive functioning; (ii) the consideration of the impact of depressive symptoms in the analyses, since depression can manifest with cognitive deficits or accentuate cognitive impairment [35]; (iii) the assessment of depressive symptoms with GDS being an instrument less susceptible to potential bias stemming from the presence of somatic symptoms in the absence of depression; (iv) the inclusion of a group of patients with MiND, characterized by cognitive dysfunction impairing performance on complex activities of daily living [22], so that the clinical significance of the detected lower cognitive performance in patients with RA or SSc becomes readily evident.

In patients with RA, performance on verbal short-term memory, verbal fluency, concentration/attention and MMSE was significantly lower compared to that of individuals without cognitive impairment, while differences in inductive reasoning and COGTEL + tended to reach statistical significance according to the final selected regression models, which included age, education and sex and independent variables. Despite the previously prevailing attitude to the absence of cognitive deficits in RA [11], mounting recent scientific evidence points to lower performance on attention, verbal fluency, logical memory, short-term memory and working memory of patients with RA [4]. Our findings are in line with previous reports, even though no general agreement regarding the cognitive domains that are affected in RA has been reached yet. The magnitude of cognitive deficits in RA is highlighted by the fact that the severity of impairment of verbal fluency and of global performance as mirrored in MMSE total scores was higher in RA than in MiND and the deficits in short-term memory were almost similar between the two groups, while concentration/attention and inductive reasoning were impaired only in RA and not in MiND (Table 2). DAS28, a marker of RA disease activity, and treatment with steroids significantly and inversely correlated with performance on several cognitive domains pointing to a direct link between disease activity/treatment with steroids and impaired cognitive function. The pathophysiology of cognitive deficits in RA seems to be shaped by a plethora of biological and clinical factors [5]. Cardiovascular complications, chronic pain, depressive symptoms as well as autoimmune and inflammatory factors, alterations in hormone levels, side effects of drugs, such as steroids, and genetic risk factors may all be involved in the pathogenesis of cognitive impairment in RA. Interestingly, there are overlaps between brain regions affecting cognitive function and pain modulation (e.g., anterior cingulate cortex, prefrontal cortex) [5].

According to the results of the final selected regression models, SSc pertains to worse performance on verbal fluency and MMSE compared to cognitively healthy individuals. It is noteworthy that verbal fluency performance in SSc was lower compared to patients with MiND and better compared to RA. The past few reports focused on SSc cognitive function point to lower performance on memory, verbal fluency, impaired attention, working memory, visual-spatial abilities, executive functions (abstraction, planning, response inhibition and set-shifting), albeit inconsistently [9–11]. Here, lower performance only on verbal fluency and not on other cognitive domains was detected in patients with SSc, even though performance on different cognitive domains was assessed. It is noteworthy that disease severity, as mirrored in SHAQ scores, and treatment with vasoreactive agents were inversely related to performance on several cognitive domains in our sample pointing to the linkages between cognitive function and the SSc- severity and treatment. The pathomechanism of impaired verbal fluency in SSc may be caused by a compromise of cerebral haemodynamics due to vaso-occlusive disease, at the level of large intra- and extracranial arterial vessels, to which SSc cognitive deficits have been previously attributed [9, 10]. On the other hand, brain vascular changes (e.g. white matter hyperintensities, vasculopathy, cerebral calcification) [7], chronic pain, drug side effects, inflammatory and biological factors, as well as the psychological burden of living with a chronic progressive disease may embody non SSc specific interacting variables that might synergistically lead to lower cognitive performance [7, 11].

Depressive symptoms were found to associate with performance on several cognitive domains. Depressive symptoms were inversely related to performance on tasks assessing prospective memory, working memory, verbal fluency, attention/concentration, as well as with MMSE- and COGTEL + total scores. GDS scores tended to be inversely associated with inductive reasoning performance. Interestingly, in RA the positive association between GDS- and DAS28 reached statistical significance. Depressive phenotypes are closely linked to cognitive impairment [36]. Common causes of depression and cognitive decline in later life have been depicted [36]. Cardiovascular risk factors, dysregulation of the hypothalamic–pituitary–adrenal axis, inflammatory processes, depression either as a risk factor for cognitive decline or as a prodromal phenotype of brain degenerative diseases are hypotheses thoroughly discussed within the frames of the ongoing debate [37].

The discrepancy between the lack of differences in depressive symptoms between adults without cognitive deficits and patients with either RA or SSc in our study and past reports [38, 39] may be attributed to differences between studies in sample characteristics, in the employed instruments for ascertaining depressive symptoms as well as to selection bias. The detected differences in cognitive performance between study groups may point, at least to some extent, to the sample size sufficiency of the present study. In addition, differences in age, in disease duration and other disease characteristics, considering the progressive character of both RA and SSc, as well as in treatment strategies may explain the discrepancy. It is noteworthy that patients with MiND and individuals without cognitive impairment were recruited and assessed during the COVID-19 pandemic crisis, which has been shown to affect mood [40, 41]. Thus, it can be reckoned that the effects of COVID-19 crisis on the mood of participants without cognitive impairment and patients with MiND may have masked differences in depressive symptoms between these groups and patients with RA and/or SSc, who were assessed prior the outbreak of the pandemic crisis. Nonetheless, GDS scores in patients with RA or SSc do not point to the presence of clinically significant depressive symptoms [27]. Thus, the clinical significance of potential differences in GDS scores between the groups would have been marginal. Of note, no differences were detected between the groups regarding treatment with antidepressants or not in our study (data not shown). In addition, several depression scales as for example the Patient Health Questionnaire-9 emphasize somatic symptoms of depression like tiredness, fatigue or lack of energy, which characterize the clinical phenotypes of RA and SSc even in the absence of depression. Thus, the use of such instruments may have resulted in an overestimation of depressive symptoms in patients with RA or SSc in previous reports. The here employed depression instrument, developed for detecting geriatric depression, puts less emphasis on somatic symptoms of depression, which are highly prevalent in older adults independently of the presence of depression or not [27, 34].

Patients with MiND were found to tend to encounter more depressive symptoms compared to individuals without cognitive impairment and patients with either RA or SSc. Depressive symptoms have recently attracted attention as parts of the neuropsychiatric symptoms that shape the clinical phenotype of MiND [42]. Despite the wide range of prevalence of depression in MiND because of the different definitions of oligosymptomatic AD, depression instruments, and diagnostic criteria employed in the different studies, the prevalence of depression in patients with MiND seems to exceed 32% or even reach 50% [43]. Interestingly, depression may embody a possible predictor of progression from MiND to dementia [42]. Thus, the detected trend of patients with MiND to suffer from more depressive symptoms compared to the other study groups is not unexpected.

The present study has several limitations. First, the size of each diagnostic group was relatively small. Nonetheless, differences in performance on several cognitive domains between the groups attained statistical significance. Second, several factors which had been shown to pertain to the presence of cognitive deficits and/or depressive symptoms in rheumatic diseases (e.g., white matter hyperintensities, vasculopathy, cerebral calcifications, cerebral hypoperfusion, carotid artery intima media thickness, c-reactive protein) [7] were not taken into account. Third, even though the study sample was not restricted to people aged 65 or older, depressive symptoms were tapped with GDS, which is a tool designed to assess depression in older adults. Nevertheless, GDS was recently shown to have good diagnostic sensitivity and specificity in detecting depressive symptoms even in adults aged 18–54 [44]. Fourth, the potential bias stemming from the temporal deviation in the assessment of the study groups and the effects of COVID-19 crisis on cognitive function and mood should be taken into account [40, 41, 45]. Nevertheless, the performance of patients with RA or SSc on several cognitive domains was lower than that of cognitively healthy individuals, while the impact of the diagnostic status of RA and/or SSc on cognitive function in many cases exceeded that of MiND (e.g. verbal fluency). Furthermore, the cognitive assessment did not include computerized testing which is superior to conventional cognitive tests for instance in terms of precision measurement of required time or reaction time [46].

## Conclusions

Compared to cognitively healthy individuals, the clinical phenotype of RA is related to worse functioning in verbal short-term memory, verbal fluency and concentration/attention, while that of SSc is linked to pooper performance on verbal fluency tasks. Of note, in both RA and SSc verbal fluency performance was lower than in MiND. Thus, the clinical significance of low cognitive function in RA and SSc becomes evident and warrants further investigation in larger samples, so that light is shed not only on the cognitive domains that are affected in RA and SSc and should possibly be regularly screened when these diseases are diagnosed, but also on the pathogenesis of these deficits, in order to develop adequate therapeutic strategies.

## Data Availability

The datasets used and analysed during the current study are available from the corresponding author on reasonable request.

## Abbreviations

RA: Rheumatoid arthritis
SSc: Systemic sclerosis
MiND: Mild neurocognitive disorder due to Alzheimer’s disease
DAS 28: Activity Score 28
SHAQ: Scleroderma-Specific Health Assessment Questionnaire
VASs: Visual analogues scales
ILD: Interstitial lung disease
COGTEL+: Cognitive Telephone Screening Instrument plus (COGTEL+)
SST: Serial Seven Test (SST)
MMSE: Mini Mental State Examination (MMSE)
GDS-15: Geriatric Depression scale-15
DSM-5: Diagnostic and Statistical Manual of Mental Disorders, Fifth edition
ANOVA: One way analysis of variance
